# Delivering person-centred dementia care: Perceptions of radiography practitioners within diagnostic imaging and radiotherapy departments

**DOI:** 10.1177/14713012231189061

**Published:** 2023-07-14

**Authors:** Robert Higgins, Adam Spacey, Anthea Innes

**Affiliations:** School of Health and Society, 7046 University of Salford, Salford, UK; 3710 McMaster University, Hamilton, ON, Canada

**Keywords:** diagnostic imaging, radiotherapy, care partners, dementia friendly, communication

## Abstract

**Introduction:**

Despite abundant literature on the diagnosis of dementia, limited research has explored the lived experiences of radiography practitioners when providing care to people living with dementia in the department.

**Objectives:**

This qualitative study explored the perceptions and compatibility of current professional guidance by both diagnostic imaging and radiotherapeutic radiography practitioners as well as the key stakeholders involved with developing the Society and College of Radiographers clinical practice guidelines for caring for people with dementia.

**Methods:**

This was a two-phase multi-method study. Fifteen diagnostic imaging and two therapeutic radiography practitioners from across the UK participated with online focus group discussions. Four key stakeholders involved with the development of the Society and College of Radiographers guidelines took part with individual semi-structured interviews. Data analysis included narrative and thematic analysis.

**Results:**

Participants from both phases identified enablers and barriers to providing person-centred dementia care. Three superordinate themes were identified linked to (1) Working with care partners, (2) Departmental environmental design, and (3) Communication and interprofessional infrastructure.

**Discussion:**

Many radiography practitioners still feel unprepared when caring for people living with dementia despite the clinical practice guidelines. Care partners were identified as having the potential to help alleviate some of the challenges radiographers faced. Participants were also aware of the impact of the departmental environment and recognised that poor way finding designs could lead to frustration. Radiography practitioners were not always aware that a patient was living with dementia prior to their attendance in the department making it difficult for practitioners to make appropriate accommodations such as additional time at appointments or the departmental environment. Our findings suggest there is a need for profession specific education and training for radiography practitioners to support the provision of person-centred dementia care. There is also a need to support the design of dementia friendly diagnostic imaging and radiotherapy departments.

## Introduction

People living with dementia may attend radiography service providers for a variety of reasons including diagnostic imaging for differential diagnosis as well as for injuries and pathologic conditions. They may also attend radiotherapy departments for co-morbid cancer treatment (Chang et al., 2016; Griffiths et al., 2015). It is estimated that approximately 850,000 people are currently living in the United Kingdom (UK) with dementia and given this number is projected to increase this will likely place a further strain on radiography services across the UK (Alzheimer’s Research UK, 2021; The Society and College of Radiographers (SCoR), 2020). Regulation in the UK requires that all healthcare professionals, including radiography practitioners, provide person-centred care (Care Quality Commission (CQC), 2014).

The philosophy of person-centred care is essential for good dementia care and is built around the needs of the individual and contingent upon knowing the person through an interpersonal relationship. It challenges the traditional medical model of care that tends to focus on processes, schedules, staff and organisational needs (Fazio et al., 2018). Brooker (2004) outlined four key components that are integral to a person-centred care approach for people living with dementia which include (a) valuing and respecting persons with dementia and those who care for them; (b) treating people living with dementia as individuals with unique needs; (c) seeing the world from the perspective of the person living with dementia, so as to understand the person’s behaviour and what is being communicated; and (d) creating a positive social environment in which the person living with dementia can experience relative well-being through care that promotes the building of relationships. Brooker (2006) expanded upon these components and identified key indicators or practices for each of the four components which included communicating effectively, treating individuals with respect and supportive and inclusive physical and social environments.

There are two branches of radiography: Diagnostic and Therapeutic. Although there are similarities between diagnostic and therapeutic radiography practitioners such as the use of specialist technology and the delivery of person centred care, there are some notable differences. For example, diagnostic radiographers produce medical images using a range of modalities and may typically have limited contact time with patients, whilst therapeutic radiographers treat patients with cancer using radiation and are involved with the patient from planning to treatment stages (College of Radiographers, 2022). However, clinical protocols used to treat and care for people living with dementia vary, resulting in inconsistent training or guidelines for health professionals which may potentially compromise the quality of care provided to people living with dementia when attending diagnostic imaging or radiotherapy service providers (Chang et al., 2016). This may have implications for radiographic practice and the delivery of person-centred dementia care. Diagnostic and therapeutic radiography practitioners have distinct different roles - diagnostic radiographers produce and process images of body structures to support a diagnosis or guide direct interventional treatments, procedures and therapies, whilst therapeutic radiographers treat patients with cancer using radiation and will be planning process, treatment and eventually post-treatment review (follow-up) stages – there are similarities in that both use specialist technology and are patient-focused.

For example, when arriving in a diagnostic imaging department, people living with dementia may exhibit behaviours that radiography practitioners find to be unpredictable (Chang et al., 2016). Frustration, along with an inability to comply with or understand instructions when undertaking diagnostic imaging procedures, can manifest as aggressive behaviour, making it difficult for the radiographer to provide adequate care or establish and sustain rapport, especially as diagnostic imaging can last between 15 and 30 min (Chang et al., 2016; SCoR, 2020). Although policy and research in this area is growing there is still a gap in the evidence base reaching practice where it is needed with many radiography practitioners reporting feel grossly underprepared when caring for people living with dementia (Miller et al., 2019; SCoR, 2020; Smith et al., 2019).

Key findings from the existing literature have focused on professional attitudes towards dementia and identified that people living with dementia can receive poor patient care or negative experiences when attending radiography services (Booth et al., 2017; Challen et al., 2018; Chang et al., 2016; Kada, 2009; Miller et al., 2019; SCoR, 2015; SCoR, 2020; Wolf & Gunderman, 2020). Despite the current clinical practice guidance produced by the SCoR and the recognition of the importance of the patient experience of people living with dementia and their care partners when undergoing diagnostic imaging and/or radiotherapy procedures little research has explored the lived experiences by radiography practitioners when caring for people living with dementia and their interactions with care partners.

The aim of this study was therefore to explore the recounted experiences of both diagnostic and therapeutic radiography practitioners in the UK when providing care for people living with dementia. It also sought to investigate the compatibility of these findings with the perspectives of key stakeholders involved in developing the SCoR ‘Caring for people with dementia’ guidelines (SCoR, 2020).

## Methods

### Design

This was a multi-method, two phase (online focus groups followed by individual semi-structured interviews) qualitative research study. An inductive qualitative approach was taken as the study sought to explore the recounted experiences and perspectives by participants.

### Sampling and recruitment

Phase 1 used purposive sampling to recruit Health and Care Professions Council (HCPC) registered qualified diagnostic and therapeutic radiography practitioners of any professional background/role based on the College of Radiographers (CoR) Career Framework for diagnostic and therapeutic radiographers (assistant practitioner, diagnostic or therapeutic radiographer, advanced practitioner, and consultant radiographer) and years of experience from across the UK. Pre-qualification student radiography practitioners were excluded from this study. Recruitment of participants was conducted via social media and by word of mouth. Phase 2 used convenience sampling to recruit participants identified as members of the core group involved with the development of the SCoR ‘Caring for people with dementia’ guidelines (SCoR, 2020). Pre-qualification student radiography practitioners were excluded from this study.

### Consent

Two versions of Participant Information Sheets (PIS) were used. The same core information about the study was included but as the methods used to gather data were different, we were required to use different versions by our ethics committee. Each PIS explained issues relating to anonymity, confidentiality, withdrawal from study and a debriefing process should the online focus group or interview cause any anxiety. For the online focus groups anonymity was preserved by ensuring that no real names or other directly identifying information were used (Sim & Waterfield, 2019). Only on actively consenting with each phase were participants able to access the online focus groups or take part with the individual semi-structured interviews. This included agreeing to maintain the confidentiality of the other participants and to not repeat opinions outside of the online focus group.

### Ethics

Ethical approval for the study was granted by the University of Salford Ethics Committee (23/11/2020).

### Data collection and management

#### Phase 1

Health and Care Professions Council (HCPC) registered diagnostic and therapeutic radiography practitioners of any professional background/role based on the College of Radiographers (CoR) Career Framework from across the UK were asked to participate with an online focus group. This offered a solution to the challenge of arranging face to face focus groups with the geographical spread of participants and COVID-19 pandemic issues. Participants were recruited to a closed online asynchronous semi-structured online focus group that allowed participants to contribute at a time that suited them and to share common experiences using a dedicated online focus group platform (https://www.focusgroupit.com/). Demographic information was also collected on professional background and years of experience (Table 1). Questions were derived following a synthesis from published literature or research from a similar context and agreed by all authors (Table 2).

**Table 1.** Online focus group demographics.

Participant	Role	Professional background and experience
**Diagnostic radiography focus group 1**		
Participant 1	Band 5 radiographer	Qualified in 2017. Special interest in dementia care and teaching students.
Participant 2	Senior computed tomography (CT) radiographer	Qualified 2014.
Participant 3	Consultant radiographer in breast imaging	Qualified in 1979. Education lead for division of clinical services.
Participant 4	Declined to provide any information	
Participant 5	Band 5 radiographer	Qualified in 2017. Special interest in dementia care and people with special needs.
Participant 6	Training and development radiographer working in breast screening	Qualified in 2011. Interest in training and research.
Participant 7	Radiography academic	Did not state when qualified but previous specialist clinical radiographer in CT and magnetic resonance imaging (MRI) and imaged patients with dementia. Area of interest due to personal experiences with relatives.
Participant 8	Declined to provide any information	
**Diagnostic radiography focus group 2**		
Participant 1	Diagnostic radiographer – did not specify band	Qualified in 1990.
Participant 2	Consultant radiographer in breast imaging	Worked both in NHS breast screening (BSP) programme setting for approximately 13 years. Interested in clinical-based research, service development and education.
Participant 3	Consultant radiographer in breast imaging	Worked in breast imaging since 1990 and been a consultant radiographer for over 8 years. Have a special interest in breast cancer in older women
Participant 4	CT/MRI radiographer and superintendent radiographer of education and professional development	Qualified in 2005. Dementia champion for department.
Participant 5	Senior radiographer/mammographer	Qualified for just over 8 years. Majority of experience in plain film and some CT. Working as a mammographer for nearly 2 years.
Participant 6	Declined to provide any information	
Participant 7	Declined to provide any information	
**Therapeutic focus group**		
Participant 1:	Advanced therapeutic radiographer in review, information, and support	Qualified in 2003. Worked in several different departments as a treatment and pre-treatment therapeutic radiographer.
Participant 2:	Therapeutic radiographer/Advanced clinical practitioner specialising in breast and non-melanoma skin cancer	Qualified in 2002. Trust dementia champion. As part of the MSc project put into place a service implementation project designed to improve the quality of care given to radiotherapy patients who are also living with dementia.

**Table 2.** Phase 1 online focus group questions.

Icebreaker/Introduction	Can you please introduce yourself by sharing a little bit about your professional background and years of experience
**Question 1**	Please describe in a few sentences what you do so that persons living with dementia and their carers get support when undergoing imaging/therapy in the department?
**Question 2**	Think about how you interact with people living with dementia and their carers when attending the imaging/therapy in the department. What are the challenges? What makes it easier?
**Question 3**	Please describe a situation (good or bad) that you have experienced when delivering care for those living with dementia or their carers when undergoing imaging/therapy
**Question 4**	What do you think people living with dementia and their carers expect from you when attending for imaging/therapy in the department?
**Question 5**	What do you think about the role of the carer in these situations?
**Question 6**	What protocols or guidelines for caring for people living with dementia have you come across in practice?
**Question 7**	Against the background of your experience what would help you to better support people living with dementia and their carers when undergoing imaging/therapy in the department?
**Question 8**	Are there any existing programmes in place in your place of work to help develop and improve your care for those living with dementia and their carers, e.g. training programmes?
**Question 9**	What do you think should be done (if anything) to improve the delivery of care for people living with dementia undergoing imaging/therapy in the department?
**Question 10**	Is there anything that should have been discussed, but not mentioned here?

Fifteen diagnostic radiographers were recruited and divided into 2 homogenous focus groups (8 and 7 participants respectively). This was to reflect the contextual differences between therapeutic and diagnostic radiography practitioners and allow common experiences to be shared and built upon. Only 2 therapeutic radiographers took part. However, the data gained from this focus group was found to be in-depth and sufficient for the objectives of this study. Author 1 acted as moderator and monitored each online focus group as well prompting any further discussions. Each online focus group ran for 4 weeks and closed at the point of data saturation (Saunders et al., 2018).

#### Phase 2

Participants from the group who developed the caring for people with dementia guidelines (SCoR, 2020) were invited to participate with a 60-min audio-recorded individual semi-structured interview via Microsoft Teams. The interview questions were inductively derived following analysis from the phase 1 data and agreed by all authors (Table 3). Participants included 1 core member of the stakeholder group and 3 members from the individual stakeholder group represented by 2 diagnostic radiographers and one therapeutic radiographer.

**Table 3.** Phase 2 individual interview semi-structured questions for stakeholders.

**Question 1**	What do you think people living with dementia and their carers expect from radiography practitioners when attending for imaging or radiotherapy?
**Question 2**	What do you think might be the barriers (if any) in how radiography practitioners provide patient centred care for PLWD?
**Question 3**	What do you think might be the barriers (if any) in how radiography practitioners and involve the carers for PLWD when attending imaging or therapy departments?
**Question 4**	What do you think might be enablers to help radiography practitioners provide a patient centred care for PLWD when attending imaging or radiotherapy departments?
**Question 5**	What do you think might be enablers help radiography practitioners involve carers for PLWD when attending imaging or therapy departments?
**Question 6**	What do you think about a carer policy to help provide advice and guidance for radiography practitioners when involving carers in the support of PLWD?
**Question 7**	What do you think about the idea of dementia leads in imaging and therapy departments?
**Question 8**	What would you consider to be a supportive environment for PLWD?
**Question 9**	What learning do you think radiography students need when preparing to care for PLWD?
**Question 10**	What do you think are the training and education needs for qualified radiography practitioners?
	Any further comments or items not discussed that you would like to raise?

### Analysis

For the online focus group data, a narrative approach was used to analyse and contextualise the data to help interpret and make sense of the participant recounted experiences or interpretation of events. This is also allowed broad ‘themes’ to be identified linked to their experiences when caring for people living with dementia (De Fina & Georgakopoulou, 2015). This was initially performed by author 1 and was then reviewed by authors 2 and 3 allowing cross-checking. These stories were then used to help develop the interview questions for phase 2. Data for both phases was also transcribed verbatim and then analysed by author 1 who systematically coded the data across the entire dataset for both phases of the study following the six-steps of thematic analysis outlined by Braun and Clarke (2006) (Table 4).

**Table 4.** Braun and Clarke’s (2006) six-phase approach to thematic analysis; including detail of how this was implemented and by whom.

Phases	Application of the phases within this study
1. Becoming familiar with the data	*Author 1* conducted the interviews. Transcripts were repeatedly read.
2. Generating initial codes	*Author 1* coded the data in a systematic fashion across the entire dataset. All focus group and interview data that related to the study aims was coded.
3. Searching for themes	All significant patterns in the data were noted and initial table of second-order codes and quotes created. Throughout this and subsequent stages, findings were reviewed for coherence and credibility by *Author 2 and Author 3* and the raw data were regularly referred to.
4. Reviewing themes	From the initial table of significant second-order codes and discussions with, *Author 2 and Author 3* candidate themes were identified. These were then refined by repeatedly referring back to data and codes, and by creating a detailed thematic map. Candidate themes were examined to establish whether they were coherent, externally heterogeneous, and had explanatory power.
5. Defining and naming themes	Through further discussions a more parsimonious list of themes were created (*Author 1*). These were refined through peer debriefing and verification with *Author 2 and Author 3*.
6. Producing the paper	The paper was drafted, with each author writing up selected themes and feedback obtained from all authors on the narratives produced.

## Findings

Findings identified both enablers and barriers to optimising radiographic care to people living with dementia across both phases. Three superordinate themes (1) Working with care partners, (2) Departmental environmental design, and (3) Communication and interprofessional infrastructure were identified that overarched six subordinate themes: care partners as an asset, delimiters to partnership working with carers, physical space, patient wayfinding, interprofessional communication and lack of knowledge about the patient. These themes were identified across both data sets/phases and are referred to within the SCoR caring for people with dementia guidelines (SCoR, 2020). Within the presentation of findings below, the following codes have been used to denote the focus group (Phase 1) and semi-structured interview participants (Phase 2):

Phase 1:

• DR = Diagnostic Radiographer Focus Group
• TR = Therapeutic Radiographer Focus Group

Phase 2:

• Interview Participant

### Theme 1: Working with care partners

Working with care partners was commonly recognised by all participants as being central to delivering person-centred dementia care when performing diagnostic imaging or treating people living with dementia. For the purpose of this study the term care partner is a holistic term used to denote someone who provides help and support to a person living with dementia and includes, partner, relative, friend or healthcare professionals such as nursing staff or care home assistants. Most participants recognised and valued the role care partners provided by highlighting how they involved the care partner:

**Participant 1 TR:** *“They [the care partners] are key, they give so much information and support. They are great at feeding back what does and doesn't work for the person living with dementia.”*

Participants felt it was important to recognise that people living with dementia should be the main focus during diagnostic imaging or radiotherapy procedures, whilst supporting the care partner’s needs:

**Participant 3 DR1:** *“It is always important to recognise the person living with dementia as the main focus, but with support from the carers…they are the person that knows and understands them the best.”*

**Participant 2 TR:** *“We also find that carers often need just as much support as the person receiving treatment… we have found sometimes as radiotherapy continues the person living with dementia due to the radiotherapy side-effects can worsen their dementia related symptoms which can greatly impact on the carer and their relationship.”*

Several benefits of care partner inclusion were evident throughout the data analysis. For example, participants recognised that often care partners have in depth knowledge of the people living with dementia and can therefore support the radiography practitioner:

**Participant 6 DR1:** *“Utilising the carer's knowledge of how the patient can react in different situations and their ability to detect different triggers.”*

**Participant 6 DR1:** *“The carer can offer valuable information to achieve better outcomes if they know the patient well. They are able to communicate with the patient in the best way for them and can reassure and calm patients.”*

Despite these benefits, several challenges to care partner inclusion were identified by the participants, including the ability to recognise when or when not to include a care partner during diagnostic imaging or radiotherapy treatment. Specifically, participants noted that care partners could either be an asset or a hindrance depending on their relationship with the person living with dementia. For example, one participant highlighted that a care partner that has a poor relationship or had only just met the person living with dementia could hinder the provision of person-centred dementia care:

**Participant 5 DR2:** *“The carer can be the greatest asset or really hinder the process. A good carer will be able to provide information to assist you and will also help in a number of ways, to support, keep calm and know triggers. A carer who doesn't have a great rapport with the patient can cause anxiety to increase.”*

Despite this, our findings suggests that participants felt that by including care partners who have a good relationship with people living with dementia, they can assist radiographers in obtaining images or help to provide reassurance during setting up for radiotherapy to support the delivery of person-centred dementia care:

**Interview Participant 1:** *“…actually involving the carer can be the thing that means that you are successful in obtaining your images with the person and the best experience for them as well.”*

Participants noted a lack of guidance and policy regarding care partner inclusion explaining how and why carers can be included in diagnostic imaging examinations or radiotherapy treatments. Our findings suggests that the absence of any policy and procedures in this area left some participants feeling unsure of their current practice with involving care partners despite the SCoR guidelines advocating carer inclusion. This may suggest a lack of awareness, familiarity or agreement with the SCoR dementia guidelines:

**Interview Participant 2:** *“It would be nice to actually have some guidelines, a clear policy that stated how we could involve carers. And that we should involve carers, you know? Rather than seeing them…oh, you must stay outside, you’re not allowed into the x-ray room, the MRI, you know.”*

**Interview Participant 4:** *“I believe that any written advice is always beneficial, I think that when there’s sort of protocols and procedures put in place, you know where you stand with things…”*

Moreover, given the potential impact on care partners themselves, it was expressed by participants that care partners should also have an active role in the development of policy and guidelines. It was also highlighted by participants that the guidance should include a safeguarding element to support the minority of cases where care partners could potentially have a negative effect on people living with dementia when attending for diagnostic imaging or radiotherapy:

**Interview Participant 3:** *“I think within the policy though, we would need to make sure that there’s some sort of safeguarding element to it …we just need to think about that small minority of cases where the carer might also inhibit or prevent a patient with dementia thinking for themselves, talking for themselves.”*

### Theme 2: Departmental environmental design

Participants discussed the physical environment and how it was not always conductive to people living with dementia and the notion of dementia friendly environments generally. Specifically, this was linked to hospitals and the lack of adequate sign posting for people living with dementia when trying to find the diagnostic imaging or radiotherapy department and getting confused and agitated as they struggled to navigate their way to the department or got lost on the way. In response to identifying way-finding difficulties one focus group participant described solutions they had found such as:

**Participant 4 DR2:** *“creating- quiet areas that were colour coded and signed accordingly.”*

This same participant felt more needed to be done to improve signage throughout the hospital and argued that:

**Participant 4 DR2:** *“The environment is a huge issue that many staff are not aware of - how it can potentially impact on a patient. Noisy waiting areas, bright lights, people walking by could be very frightening”.*

One of the interview participants also raised issues with making departments dementia friendly:

**Interview Participant 2:** *“So things like the design of a waiting room, and the stuff on the walls, and different bits and pieces. Because it doesn’t have the look of what maybe the design team want when they’re doing a project, it gets put aside as not important.”*

Participants also discussed changes made to the environment such as a quiet room for people living with dementia:

**Participant 1 TR:** *“We have a specialist 'quiet' room which has been ergonomically designed with dementia patients in mind who prefer not to be in a busy waiting area. We have a selection of books about the past, puzzles and games such as dominoes and twiddle muffs [a knitting activity].”*

However, participants also noted that further improvement was still needed linked to the environmental design of the department such as a dementia clock and appropriate signage to help people living with dementia orientate themselves and help with telling the time:

**Participant 1 TR** *“I would also like to get a dual digital clock in the room with time and date and an orientation sign with hospital and department name along with good, clear signage”.*

Furthermore, participants stated that resources required to make the required adaptations to improve the physical environment for people living with dementia might be limited due to cost:

**Participant 2 TR** *“Finances to improve the environment for those living with dementia might be a barrier.”*

Despite Participants acknowledging the importance of dementia friendly waiting areas, they did not provide any detail of their understanding of this and what that this might entail in practice. Participants discussed the importance of other calming measures in the environment that did not require new resources, rather the adoption of careful planning and attention to the individual needs of the person living with dementia, such as music playing and adjusting the lights to provide a sense of safety and comfort. One of the diagnostic radiographer focus group participants argued that their hospital needed to go further:

**Participant 1 DR2:** *“Dementia-friendly areas (not just the addition of one coloured wall to signify an exit point).”*

This demonstrated an understanding of the tokenism by the participants that may be present when using the buzz words of ‘dementia friendly environments’ in the hospital setting. Promoting safety in the environment was also discussed with risks of absconding and falls mentioned most frequently by participants:

**Participant 1 DR1:** *“Patients absconding the department when they are not accompanied and falling from their bed when left unattended.”*

Participants raised the need to monitor people living with dementia when attending for diagnostic imaging or radiotherapy to help prevent this happening if family members were not present. Once people living with dementia reached the waiting room, and in the absence of safe, enclosed spaces one participant described measures they took to support the people living with dementia to stay in the waiting room until their appointment:

**Participant 2 TR:** *“In our department we reduce the risk of absconding by escorting people with dementia to and from treatment rooms, ensuring they are reunited with relatives. We ask receptionist, assistants and students to sit with patients who come in alone.”*

### Theme 3: Communication and interprofessional infrastructure

Good communication with people living with dementia was seen as central to person-centred dementia care along with providing privacy, dignity and respect by the participants. This was to ensure not only patient compliance with procedures but also to provide reassurance and aid patient understanding of the procedure leading to a positive outcome:

**Participant 5 DR2:** *“I always speak clearly and calmly. I make sure that I explain exactly what I am going to do before I do it. Plenty of reassurance throughout the examination is key, along with making sure that the patient feels safe and as comfortable as possible”.*

Communication with family members or care partners was also seen as being important by participants:

**Participant 4 DR2:** *“I ensure I communicate with the patient, family and carers prior to any Imaging I undertake. If the patient is an in-patient and Dementia is noted in the clinical history, I ensure I communicate with the staff on the ward... Whilst in my care, I ensure I communicate with the patient, explaining everything that is happening…”*

People living with dementia may have problems expressing language which can prove frustrating for both the person living with dementia and the radiography practitioner. This is an important consideration as dementia affects the areas of the brain that enables people to verbalise or identify the right words and to understand what is being said (Hobson, 2019). This was emphasised by participants linked to compliance or obtaining consent:

**Participant 3 DR1:** *“One of the main challenges is judging how much information the patient can understand in order to gain compliance.”*

Other factors thought by participants to impact on communication with people living with dementia included restrictions in hospitals due to the recent COVID-19 pandemic resulting in patients attending departments without carers which posed difficulties with communication:

**Participant 5 DR1:** *“When the patient attends for x-ray alone with no family member/carer... This happened a lot during COVID where patients attended from the wards on their own. Getting the patient to understand that you don't want to hurt them and that you may need to undress, touch or move them to be able to achieve a diagnostic image.”*

Interestingly, participants seemed to focus on verbal communication rather than body language when interacting with people living with dementia. As verbal skills deteriorate, other forms of communication become more significant as body language may expresses unmet needs, resulting in challenging behaviours (Social Care Institute for Excellence (SCIE), 2020) and this particularly highlighted by the focus group participant:

**Participant 1 DR1:** *“I was working in A&E doing a pelvis x-ray on a patient with advanced dementia. The patient was very agitated and flailing their arms around the trolley and shouting out for help. They did not have an escort or carer with them. The radiographers at the time were ignoring the patient and just trying to get on with taking the x-ray.”*

Participants also identified concerns linked to whether people living with dementia could consent to their diagnostic imaging or radiotherapy treatment:

**Participant 3 DR1:** *“Within breast imaging it is not uncommon for interventional procedures to be undertaken on demand and obtaining consent can present a difficulty if the patient is deemed not to have capacity to consent.”*

Issues around the lack of interprofessional communication linked to forewarning of people living with dementia when attending diagnostic imaging or radiotherapy departments were also noted by participants:

**Participant 2 DR2** *“Often radiographers are pushed for time and want to spend more time with patients but are restricted due to the workload and appointment times. If we know a patient has particular needs, not solely people living with dementia but any patient who may require support then we can allocate more time and have an extra radiographer to support the examination.”*

## Discussion

Findings from our study identified three key themes linked to both facilitators and barriers for radiography practitioners delivering person-centred dementia care which were identified across both phases of the study. These included working with care partners, departmental environmental design, and communication and interprofessional infrastructure, which have also been highlighted in the SCoR caring for people with dementia guidelines (SCoR, 2020). When discussing working with care partners participants were able to recognise and use the knowledge of care partner during diagnostic imaging or radiotherapy treatments. This was found to support the delivery of person-centred dementia care leading to better outcomes for all involved. However, there was also a perception by some participants that there could be issues based on the care partners relationship to the person living with dementia. For example, a family member may know the person living with dementia better and provide more support, as opposed to a ward nurse who may have met them only a few hours beforehand. Issues around poor care partner inclusion by radiography practitioners when caring for people living with dementia have been highlighted before, (Challen et al., 2018) but our findings would seem to indicate a positive shift in radiographers’ attitudes and understanding of care partner inclusion since 2018. Nonetheless, it was evident from our data analysis that there is need for further guidance to be implemented in relation to care partnership working to help sustain a more consistent and evidenced based practice by diagnostic imaging and therapeutic service providers.

Participants were also aware of the impact of the hospital environment on their ability to deliver person-centred dementia care and the difficulties of providing care to people living with dementia in the hospital setting is well documented (Waller & Masterson, 2015). Participants also recognised the impact of the lack of dementia friendly spaces or signage on people living with dementia wellbeing. For example, the potential for people living with dementia to become agitated before they even entered the examination room due to difficulties locating the diagnostic imaging or radiotherapy department. Although, some participants identified the term ‘dementia friendly’ and what this might mean, any improvements seemed to be curtailed by a lack of resource or spending by departments to make these changes. Information exists on how to improve acute wards for people living with dementia (e.g., Brooke & Semlyen, 2019) but the challenge lies with applying this to diagnostic imaging and radiotherapy departments with participants recognising the need to create an environment that avoided unnecessary stress and confusion for people living with dementia but felt constrained in their ability to action these ideas to improve the physical environment.

Participants suggested a way to improve person-centred dementia care would be to have more forewarning in place to identify the need for longer appointment times or time spent communicating with people living with dementia and their care partners. Several barriers were identified by participants linked to the lack of communication that occurred between people living with dementia and the radiography practitioner. However, it was recognised that this was a key element of person-centred dementia care and importance of involving care partners to support radiography practitioners when undertaking imaging or radiotherapy procedures on people living with dementia. Nonetheless, there was a focus by the participants on verbal communication rather than identifying the role of non-verbal cues when caring for people living with dementia. Whilst unusual behaviours might reflect attempts to actively express discomfort by people living with dementia, radiography practitioners may perceive these behaviours as signs of dementia (Veselova, 2013; Desai et al., 2012; Miller et al., 2019) and choose to ignore them. Interestingly, as previously noted by Miller et al., (2017) participants described generic characteristics linked to dementia when recounting their experiences, rather than noting any differences in ‘early’ and ‘later’ stages of dementia during their discussions.

Another challenge identified by the participants concerned the lack of any forewarning linked to cognitive impairment given in advance to help them prepare or make any adjustments for people living with dementia when attending for imaging or radiotherapy service providers. For example, referral requests made by general practitioners (GPs) or medical professionals tended to focus on the reason for referral but excluded information such as making them aware if the patient was known to be living with dementia which restricted any flexibility with providing person-centred dementia care such as longer appointment times or times to avoid sundowning to prevent people living with dementia feeling confused or agitated when attending for imaging or radiotherapy (Dementia UK., 2021). The SCoR caring for people with dementia guidelines (SCoR, 2020) recommend that there should be a question on diagnostic imaging and radiotherapy referral forms that includes a request for information about a person’s type of dementia and cognition, but our findings may suggest that this is not happening in practice.

Radiography practitioners clearly recognised the importance of adapting and providing person-centred dementia care, but our study would seem to suggest there is still a gap in knowledge or training in how to deliver this despite radiography professional guidelines. However, sustaining this type of dementia training may be difficult in a busy department and currently there no role specific tailored training for radiography practitioners linked to delivering person-centred dementia care in the workplace, rather there is currently a reliance on generic materials which may not link theory with radiography practice or workplace experiences (Higgins et al., 2022, 2023).

### Limitations

Despite this being a UK nationwide study, recruitment to phase 1 was lower than expected. However, the wide geographical spread of participants supports the representativeness of experience and practice. Participants for phase 1 may have self-selected already having an interest in dementia. However, the study did include perspectives from a range of professional experience and backgrounds. For phase 2 wide recruitment from members of the core stakeholder group was not achieved but nonetheless the data acquired can still be considered relevant in relation to the study aims. It is also important to recognise that social desirability bias may have affected the findings by participants trying to present a positive self by not disclosing certain thoughts or perceptions to avoid embarrassment and repercussions from others (Latkin et al., 2017).

### Further work

Future work is suggested to help identify what are areas of a dementia friendly environmental design have the potential to reduce the incidence of agitation by people living with dementia. By identifying what relatively straightforward or inexpensive changes to the design of diagnostic imaging or therapeutic departments can be made to meet the needs of people living with dementia and their carer partners may help to reduce the scepticism that making departments more dementia friendly may be an expensive, time-consuming activity. Further work is also needed to develop role specific tailored training linked to bridge the gap with radiography practitioner workplace learning rather than reliance on generic materials that are currently available to support person-centred dementia care delivery in both diagnostic imaging and radiotherapy departments.

## Conclusions

Enablers to delivering person-centred dementia care identified the inclusion of care partners, appropriate communication, and prior identification of people living with dementia as part of the referral process. Dementia friendly environments within departments that not only supported wellbeing but also allowed easy access and way finding were identified as being important in delivering person-centred dementia care. Despite the development of the SCoR caring for people with dementia guidelines (SCoR, 2020) which have been designed to support diagnostic imaging and radiotherapy service providers when caring for people living with dementia our findings suggest that many radiography practitioners may lack knowledge or feel unprepared when providing person-centred dementia care. This may in part be due to a lack of profession linked education and training in this area and recommendations include the development of role specific tailored training to bridge the gap in workplace learning rather than a reliance on the generic materials that are currently available. Other recommendations include more robust referral systems that would help to radiography practitioners make appropriate accommodations when people living with dementia attend diagnostic imaging or radiotherapy service providers and support the delivery of person-centred dementia care.
